# The impact of dietary factors on the function of brown and beige adipose tissues—implications on health and disease

**DOI:** 10.3389/fnut.2025.1626068

**Published:** 2025-08-04

**Authors:** Bruna Bombassaro, Gabriela Batitucci, Marcela Reymond Simoes, Eliana P. Araujo, Licio A. Velloso

**Affiliations:** ^1^Laboratory of Cell Signaling, Obesity and Comorbidities Center (OCRC), University of Campinas, Campinas, Brazil; ^2^School of Nursing, University of Campinas, Campinas, Brazil; ^3^Faculty of Medical Sciences, University of Campinas, Campinas, Brazil; ^4^National Institute of Science and Technology on Neuroimmunomodulation, Campinas, Brazil

**Keywords:** thermogenesis, mitochondria, food, calorie, uncoupling protein

## Abstract

The development of strategies that promote the activation of adaptive thermogenesis in brown and beige adipose tissues is expected to lead to advances in the prevention and treatment of obesity, diabetes, and cardiovascular diseases. Currently, there is a great effort to identify or develop pharmacological agents that could promote adaptive thermogenesis. However, the greatest candidates, the β3-adrenergic receptor agonists, have proven troublesome as they promote unwanted cardiovascular effects. An important advance in the field of adaptive thermogenesis was made by the identification of nutrients or nutrient-derived substances that activate thermogenesis in brown and beige adipose tissue. The detailed characterization of the mechanisms by which nutrients activate thermogenesis could lead to changes in the nutritional guidelines for metabolic diseases. In addition, these studies could unveil new targets for the development of pharmacological strategies to treat metabolic and cardiovascular diseases. In this article, we review the data that led to the current understanding of the actions of nutrients or nutrient-derived substances as thermogenic agents.

## Introduction

All living organisms are capable of producing heat, a process known as thermogenesis. Independently of the type, thermogenesis is always driven by energy, which means that organisms must metabolize fuel to heat their bodies (1). Currently, there is great scientific interest in the mechanisms of thermogenesis, as it is believed that advances in this field could lead to the development of new strategies to prevent and treat obesity and other highly prevalent metabolic and cardiovascular disorders (2).

While most organisms produce heat as a consequence of biological processes, such as cellular metabolism, exercise, and feeding, only mammals, birds, and some reptiles are capable of producing heat in response to changes in the environmental temperature (3). Three types of thermogenesis occur independently of environmental temperature: (i) obligatory thermogenesis that results from energy dissipated during cellular metabolism; (ii) postprandial thermogenesis that results from the thermal effects of food; (iii) exercise-induced thermogenesis that results from the energy dissipated during muscle contraction (3, 4). Conversely, there is only one type of thermogenesis that is activated in response to environmental cold, adaptive thermogenesis. Due to its plasticity, adaptive thermogenesis seems the strongest candidate to become a target for the prevention and treatment of obesity and comorbidities (5, 6). Thus, over the last 15 years, researchers have evaluated strategies that could safely increase energy expenditure and substrate utilization by increasing adaptive thermogenesis. Of course, pharmacological approaches are the prime focus of research in the field. However, nutrients have also been studied with a focus on their putative capacity to promote or facilitate adaptive thermogenesis. The identification of such nutrients could lead to changes in the current dietary recommendations aimed at reducing body mass and improving metabolic control. In this article, we review the advances obtained in the characterization of nutrients as agents that could promote or facilitate adaptive thermogenesis. For readers interested in a broader view of the biology and clinical aspects of thermogenesis, we recommend the reading of other authoritative reviews (7–9). Nevertheless, before entering the main subject of the article, we provide brief descriptions of the mechanisms that regulate adaptive thermogenesis.

## Brief description of the search strategy

This is a narrative review. The authors BB, GB and MRS performed the primary search for original articles, published in English and indexed in PubMed. The search had no limits on the year of publication. The search terms were: brown adipose tissue; beige adipose tissue; brite adipose tissue; BAT; brown adipocyte, beige adipocyte; brite adipocyte; thermogenesis; thermogenic; nutrient; food; and food component. All articles identified in the primary search were evaluated by the authors EPA and LAV, and only those articles considered relevant by all the five authors were included in the review.

## The control of brown adipose tissue function

**Figure 1:**
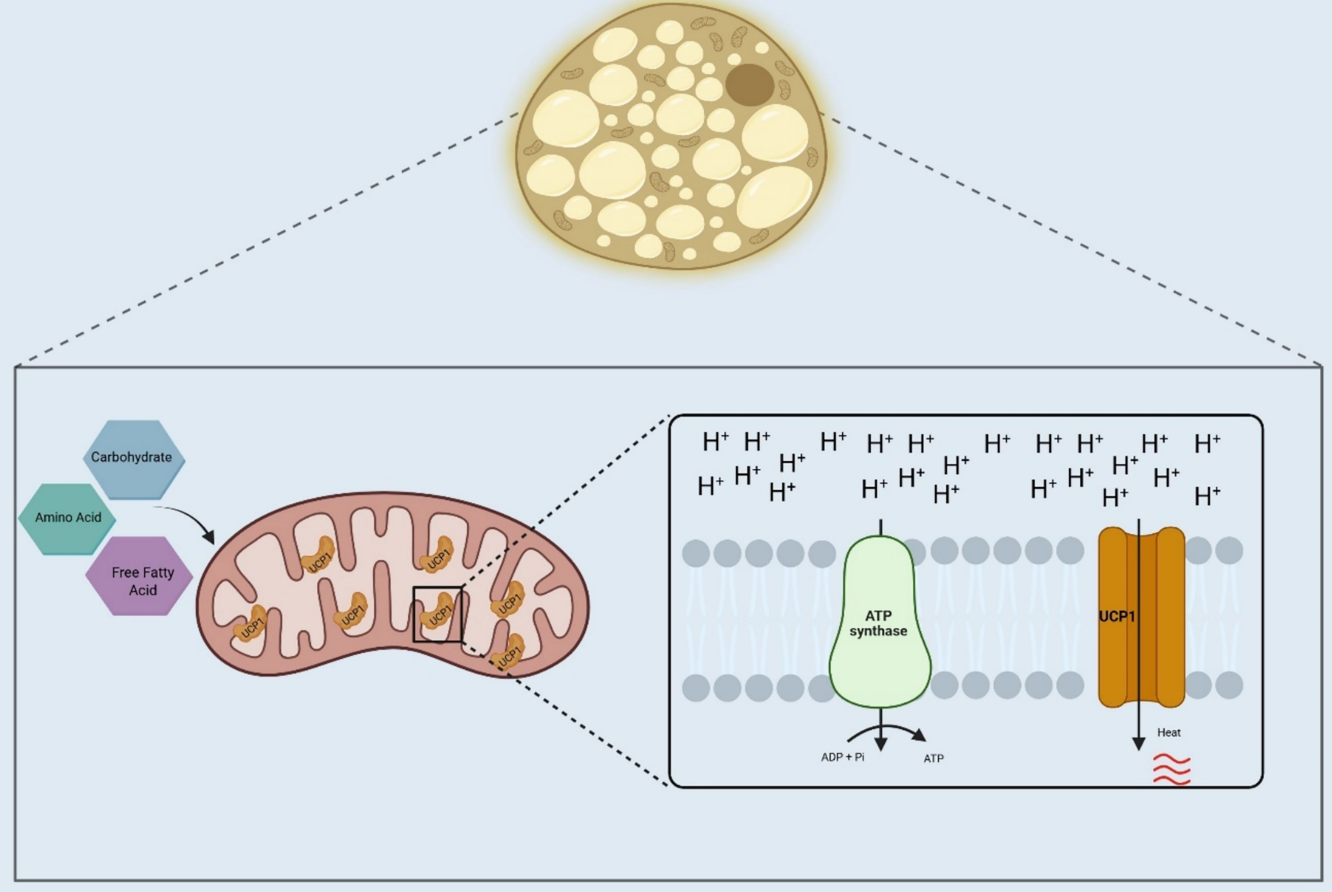
Adaptive thermogenesis in brown adipose tissue. Brown adipose tissue in humans and other mammals will use substrates, such as fatty acids, carbohydrates, and amino acids, for heat production. In the mitochondria of the brown adipocytes, the electrochemical gradient produced by nutrients oxidation will bypass the ATP synthase, which will be catalyzed by the UCP1, generating heat. UCP1, Uncoupler Protein 1; ADP, Adenosine diphosphate; ATP, Adenosine triphosphate; Pi, Inorganic phosphate; H+, Hydrogen ions. Created in BioRender. Velloso (2025) https://BioRender.com/pkgycy4.

Studies performed originally in hibernating mammals, and later in other species, including humans, have shown that the brown adipose tissue (BAT) is the main responsible for adaptive thermogenesis. It does so by employing fatty acids, carbohydrates, and even amino acids, as substrates for heat production (10). The process depends on a biological adaptation of mitochondrial respiration by which, instead of using the intermembrane electrochemical gradient to produce ATP, it is employed to produce heat (11). This is possible mostly due to the existence of an uncoupling protein (uncoupling protein-1, UCP1) that catalyzes the leak of protons across the mitochondrial inner membrane, thus, dissipating the electrochemical gradient generated by the electron transport chain (Figure 1).

**Figure 2:**
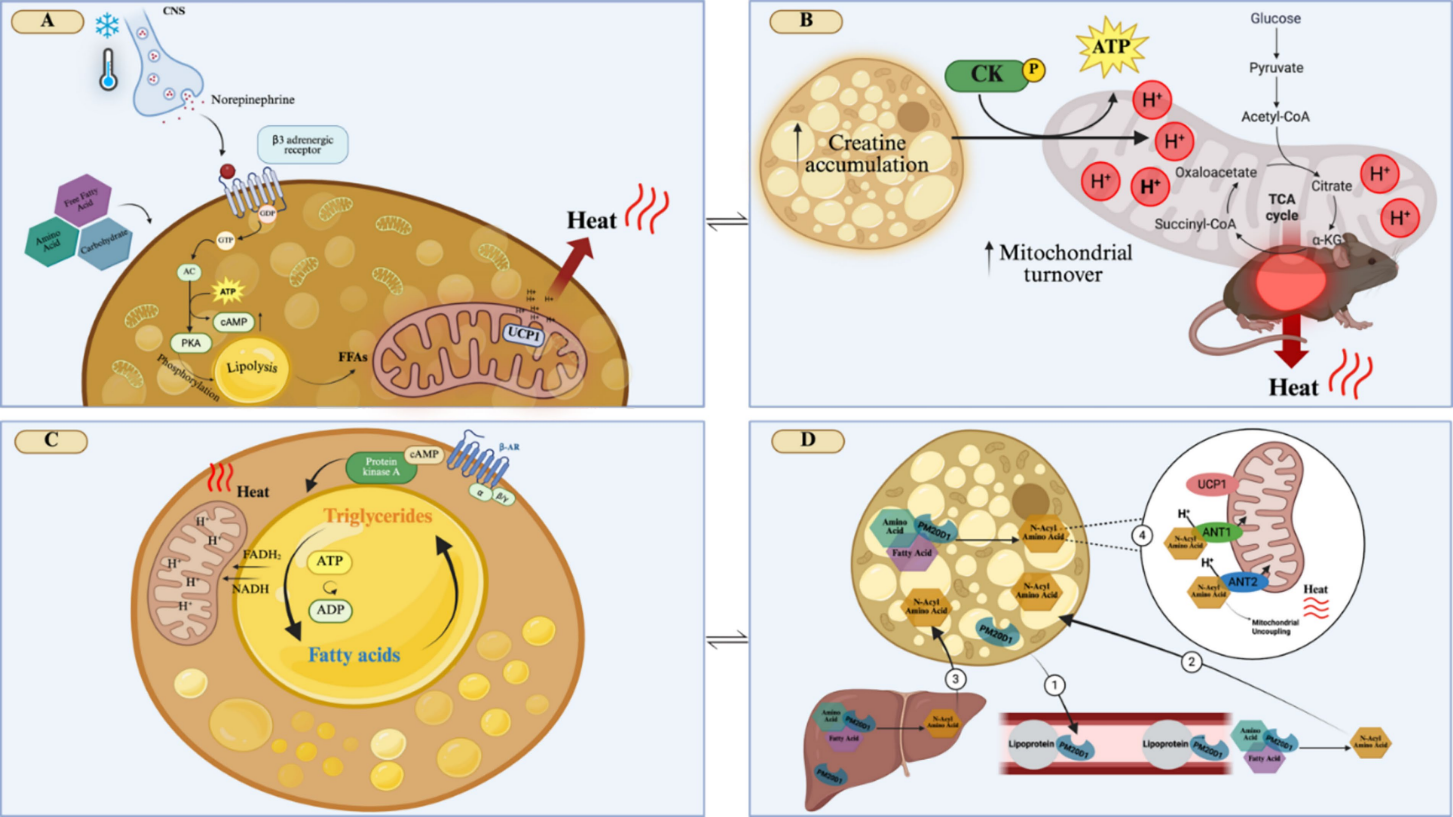
Mechanisms that activate adaptive thermogenesis in brown adipose tissue. **(A)** Cold exposure activates the release of norepinephrine by the sympathetic nervous system, which activates β3-adrenergic receptors on brown adipocytes. This will trigger a cascade of events, such as intracellular lipolysis and the expression of mitochondrial thermogenic genes, such as UCP1, leading to energy dissipation as heat. **(B)** Creatine accumulation in brown adipocytes will increase the ATP/ADP mitochondrial turnover, due to the activation of creatine kinase, which will increase the electrochemical gradient, resulting in heat generation. **(C)** Activation of β-adrenergic receptors will lead to a futile lipid cycle of simultaneous lipolysis and re-esterification of fat in brown adipocytes. Lipolysis results in the formation of FADH2 and NADH, which increases the proton gradient that contributes to energy dissipation as heat. The constant turnover of ATP/ADP and increased enzymatic intracellular activity contribute to heat production. **(D)** In brown adipocytes, PM20D1 can catalyze the condensation of fatty acids and amino acids to produce N-Acyl Amino Acid. PM20D1, which can be secreted by brown adipocytes, circulates along with lipoprotein, forming N-Acyl Amino Acid (1). PM20D1 is also secreted by the liver, being able to also form N-Acyl Amino Acids in this tissue. These N-Acyl Amino Acids can enter the brown adipocytes (2 and 3). Once in the brown adipocytes, N-Acyl Amino Acids can bind to the mitochondria, by the carriers ANT1 and ANT2, acting as endogenous uncouplers of mitochondria, independent of UCP1, generating heat (4). CNS, Central Nervous System; GDP, Guanosine diphosphate; GTP, Guanosine triphosphate; AC, Adenylate cyclase; cAMP, Cyclic adenosine monophosphate; PKA, Protein kinase A; FFA, Free fatty acid; CK, Creatine kinase; a-KG, Alpha-ketoglutarate; B-AR, B-adrenergic receptor; FADH2, Flavin adenine dinucleotide; NADH, nicotinamide adenine dinucleotide; PM20D1, peptidase M20 domain containing 1 ANT1, Adenine nucleotide translocator 1; ANT2, Adenine nucleotide translocator 2; UCP1, Uncoupler Protein 1; H+, Hydrogen ions; ADP, Adenosine diphosphate; ATP, Adenosine triphosphate; TCA, citric acid cycle; Created in BioRender. Velloso (2025) https://BioRender.com/qoh5u7x.

The canonical mechanism of BAT activation occurs when exposure to cold temperatures triggers a sympathetic response that promotes norepinephrine (NE) release from nerve terminals near brown adipocytes. NE then activates β3-adrenergic receptors in brown adipocytes, promoting lipolysis (12). Within brown adipocytes, fatty acids induce a conformational change in UCP1 leading to its activation, and thus to a heat-producing uncoupled mitochondrial respiration (12, 13) (Figure 2A).

The elucidation of the mechanism of thermogenesis induced by noradrenergic stimulus has prompted the search for pharmacological agents that could promote BAT activation, increasing energy expenditure and substrate utilization. In mice, the treatment with the β3-adrenergic agonist, CL316243, promotes BAT-induced thermogenesis, which is accompanied by reduced fat mass and increased glucose tolerance (14). In concert, in humans, a high dose of mirabegron, a β3-adrenergic agonist approved for the treatment of overactive bladder, is effective in increasing BAT activation, increasing metabolic rate, and reducing blood glucose levels (15). However, due to potent cardiovascular effects, the beneficial metabolic actions promoted by a high dose of mirabegron are accompanied by adverse properties, such as increased heart rate and increased blood pressure (16). Thus, it is currently assumed that the agonism of β3-adrenergic receptors could be potentially interesting to activate BAT in humans; however, there are at least two important questions that should be explored before more conclusive actions could be taken: (i) what are the particularities about the differences in identity, function and anatomical distribution of β3-adrenergic receptors in mice and humans (17)?; (ii) is there a possibility of producing β3-adrenergic agonists that act safely in the BAT and lack adverse cardiovascular effects?

As the safe pharmacological activation of BAT thermogenesis using β3-adrenergic agents remains elusive, other candidates are under investigation. A great advance was obtained following the unexpected observation that mice lacking UCP1 retained some adaptive thermogenic capacity (18). The detailed characterization of this phenomenon led to the identification of UCP1-independent adaptive thermogenesis. One such mechanism is dependent on creatine accumulation in the brown adipocytes (19). Under this condition, the mitochondrial turnover of ATP/ADP is accelerated, which could occur through the activation of creatine kinase activity or creatine-dependent spatiotemporal buffering of ATP (20). Independent of the mechanism, the result is the leakage of protons from the mitochondrial intermembrane space, producing heat (Figure 2B). In mouse models, the supplementation with 2% creatine (weight/volume) during an HFD protocol, reduced obesity, improved insulin sensitivity and also increased the expression of thermogenic genes in BAT (21). In vegetarian young adult humans (18–30 years), supplemented with 0.03% (weight) of creatine there was no increased BAT activity after cold exposure (22). This highlights the importance of more studies on creatine supplementation to provide a further understanding of its potential impact on improving thermogenesis in humans.

A futile cycle of lipolysis/re-esterification has been proposed as yet another mechanism of UCP1-independent thermogenesis. Brown adipocytes are equipped with enzymatic machinery capable of breaking down and re-esterifying fat. In this cycle, the re-esterification of fatty acid with glycerol acts as a sink for ATP, releasing energy as heat (23) (Figure 2C). A third mechanism of UCP1-independent thermogenesis relies on sarcoplasmic/endoplasmic-reticulum calcium cycling. As this mechanism was described in beige adipose tissue, details will be presented in the following section.

An interesting advance in the field was obtained by the description of the thermogenesis-inducing activity of n-acyl amino acids (24). This mechanism depends on the expression of the secreted enzyme peptidase M20 domain containing 1 (PM20D1) by brown and beige adipocytes, as well as other organs, such as the liver. Once secreted, PM20D1 catalyzes the condensation of fatty acids and amino acids to produce n-acyl amino acids (Figure 2D). N-acyl amino acids are produced endogenously by brown and beige adipose tissue or are produced by other tissues and blood and can enter the adipocytes. In the adipocytes, these molecules bind to the mitochondria and act as endogenous uncouplers, independently of UCP1 (24). In a recent study, it has been shown that this mechanism explains, at least in part, differences in thermogenesis among mouse strains frequently used in biomedical research (25).

As research advances in the field, it becomes clear that uncoupled respiration leading to thermogenesis can occur by different mechanisms, thus opening a wide window of opportunities for the search of potential targets to stimulate BAT as an intervention to prevent and treat obesity-associated metabolic and cardiovascular diseases.

## The control of beige adipose tissue function

In 2012, Bruce Spiegelman’s group identified another type of adipose tissue that occurs within areas of white adipose tissue and originates from the trans-differentiation of white adipocytes into brown adipocyte-like cells (26). This tissue is mostly referred to as beige, but in some articles, it appears as brite adipose tissue. In the original study describing the existence of beige adipocytes, it was shown that exercise induces the expression and secretion of the hormone irisin from skeletal muscle cells (27). Circulating irisin binds to TRCP3 receptors in adipocytes triggering the ERK and AKT signaling cascades that engage PPARγ leading to the transcriptional induction of UCP1 expression. Further studies revealed that the process of trans-differentiation of white to beige adipocytes is very dynamic and two-handed (28). This means that whole body metabolism can be improved under certain conditions by increasing the mass of beige adipocytes; however, it implies that even in organisms containing a large mass of beige adipocytes, periods of life under non-healthy conditions, such as sedentariness and ingestion of large amounts of fats and sugars, can promote the reversal of trans-differentiation of beige adipocytes back to white adipocytes.

Much less is known about the biology of beige adipocytes as compared to brown adipocytes. This is particularly relevant considering that the identity details of beige adipocytes in humans are still under investigation. The supraclavicular BAT depots present in adult humans are highly thermogenic and in early studies, it was believed it was similar to the rodent BAT; however, it is currently known that its transcriptional signature is closer to the one found in mouse beige adipocytes as compared to mouse BAT (29, 30). In addition, it is still unknown if the rodent beige inguinal depots or BAT adipose tissue faithfully represents human thermogenic adipose tissue, as there are several distinctions between them. As an attempt to address this issue, it was proposed that human thermogenic adipose depots could be modeled by interscapular BAT from humanized mice kept at thermoneutrality and fed a high-fat diet (31). However, this model is rather complex, thus, it remains unclear how representative this protocol is for human thermogenic adipose tissue. Nevertheless, in mice the distinctions between BAT and beige are clearer; as beige adipocytes derive from white adipocytes, it means that their embryonic development is different from the brown adipocytes that originate from Myf5 + cells, as it occurs in skeletal muscle development (32). Nevertheless, most transcriptional and epigenetic regulators operate similarly in the development and activation of brown and beige adipocytes. This is true for PDGFRα, EBF2, BMP7, and PRDM16 (33). Conversely, there are a few factors that act exclusively on beige adipocyte differentiation, such as MRTFA and KLF11 (33). Understanding how the small transcriptional differences between the two types of thermogenic tissues work could lead to the identification of new potential targets for the development of thermogenic drugs.

As a rule, beige adipocyte thermogenesis is mostly dependent on the expression and activation of UCP1. However, studying mice with a white adipose tissue (WAT)-selective knockout of UCP1, Shingo Kajimura’s group identified a novel UCP1-independent mechanism of mitochondrial respiration uncoupling that leads to a cold-induced thermogenic response. This mechanism, which also is found in BAT, results from the SERCA2b-dependent calcium cycling that promotes not only thermogenesis but also, increased glucose consumption (34).

**Figure 3:**
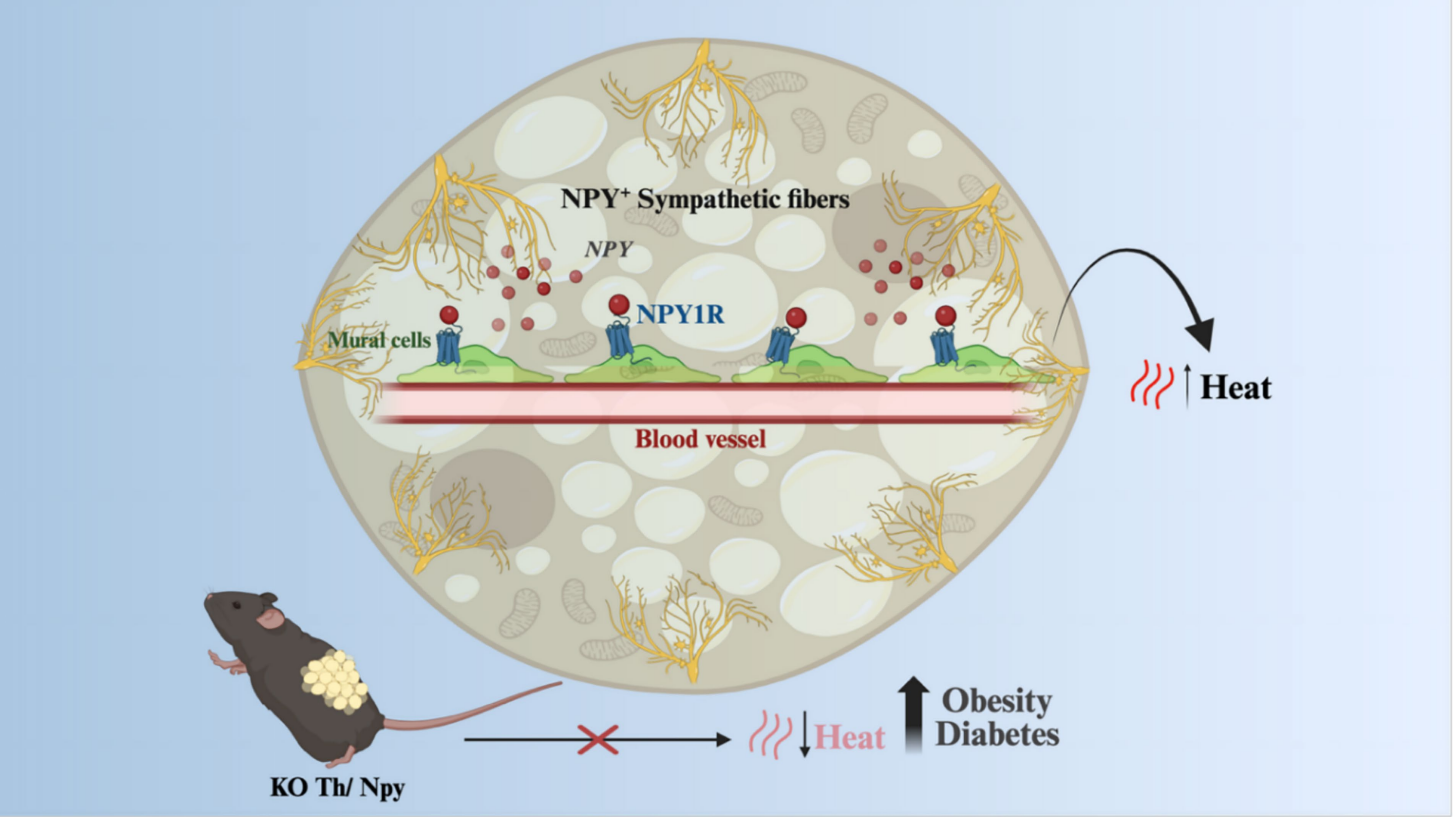
Beige and brown adipocytes response to sympathetic signals. NPY, co-expressed with noradrenaline in sympathetic fibers in response to stimuli such as increased sympathetic tonus, can promote the proliferation of iWAT and iBAT mural cells, which are progenitors of thermogenic adipocytes. Animals with a knockout for NPY in sympathetic fibers had a decrease in adaptive thermogenesis, becoming more susceptible to obesity and diabetes. KO, Knockout; NPY, Neuropeptide Y; NE, norepinephrine; NPY1R, Neuropeptide Y receptor Y1; TH, Tyrosine hydroxylase. Created in BioRender. Velloso (2025) https://BioRender.com/qvwi364.

In addition to irisin, beige adipocytes also respond to the sympathetic signals induced by exposure to cold, such as occurs in BAT. In this context, a recent study has provided important advance in this field demonstrating that, whereas NE is important to induce uncoupled respiration, NPY, which is co-expressed with NE in 32% sympathetic fibers, promotes the differentiation of beige and brown adipocytes from perivascular mural cells (35). This has proven a very important mechanism of activation of thermogenesis as mice lacking NPY in sympathetic fibers are obesity-, and diabetes-prone (Figure 3).

Another important aspect of beige adipocyte biology is the apparent capacity of activation by PPARγ agonists. Thus, rosiglitazone has been shown to promote beige adipocyte differentiation in experimental models and isolated cells (36, 37). However, in humans, studies are controversial. In primary human adipocytes, the thiazolidinediones are capable of inducing a thermogenic program. However, in living humans, the treatment with pioglitazone for 6 months was incapable of producing significant changes in the BAT (38).

Because of the greater plasticity of beige adipose tissue as compared to BAT, it is currently believed that developing interventions capable of increasing the mass of beige adipocytes may result in important advances in the strategies to prevent and treat obesity-associated metabolic and cardiovascular diseases. In this context, nutrients have emerged as important players.

## Nutrients and food components that modulate brown and beige adipose tissue functions

Most research in the field has evaluated the thermogenic capacity of food components, instead of nutrients, and most of this work was performed in experimental models. Nevertheless, some interesting data came from clinical studies. Here, we will first look at the data on food components evaluated in experimental studies and human studies. Next, we will look at the data on nutrients in experimental studies and clinical studies.

### The evidence for the thermogenic actions of food components in experimental models

In early studies, caffeine was shown to produce a thermogenic response in lean (39) and obese (40) rats. In the study evaluating lean rats, the thermogenic response was shown to depend on BAT activation (39), whereas, in the study evaluating the obese rats, there were no experiments aimed at looking at BAT, however, respirometry determinations revealed increases of up to 50% in O_2_ consumption. In mice, the thermogenic effect of caffeine was shown to occur in non-genetic obesity, whereas in the monogenic *ob* and *db* mice, its effect was minimal (41). Most caffeine actions to promote BAT thermogenesis depend on intact sympathetic innervation and are due to its activity in the central nervous system antagonizing adenosine and inhibiting phosphodiesterase activity (42). An important aspect of caffeine-induced thermogenesis in rodents is that it can enhance exercise-induced thermogenesis (43). In addition, caffeine was shown to improve glucose metabolism, systemic inflammation, and hepatic steatosis in an animal model of diet-induced obesity. Thus, in experimental models, the thermogenic and metabolic actions of caffeine are scientifically sound.

Capsaicin and capsinoids, the spicy compounds found in chili peppers, are food components that have been extensively studied for their thermogenic actions. In rats, it was shown to produce BAT thermogenesis, which was accompanied by increased GDP binding and mitochondrial O_2_ consumption (44). In mice fed a high-fat diet, capsaicin reduced adiposity and increased ATP-dependent activation of thermogenic gene expression. This outcome was due to ATP-consuming calcium and creatine futile cycles with increased expressions of SERCA2, ryanodine receptor 2, creatine kinase B, and creatine kinase mitochondrial 2, and was mediated by the combined activation of β3-adrenergic receptor, α1-adrenergic receptor, and TRPV (44). Different from caffeine, which has predominantly central actions that activate BAT thermogenesis by sympathetic connections, capsaicin promotes thermogenesis by both central and direct actions upon the brown adipocytes (45, 46).

Observational data suggested that cinnamon could act as an anti-diabetic agent (47), which raised interest in this food component as a metabolically interesting intervention. In an experimental mechanistic study, it was shown that, at least in part, the metabolic benefits leading to improved glucose tolerance were due to the effects of cinnamon to increase BAT UCP1 expression (48). The main bioactive component of cinnamon is cinnamaldehyde (49). In rats, the treatment with cinnamaldehyde reduced white adipocyte hypertrophy and the expression of genes involved in lipogenesis, Srebf1c, and Acaca, while stimulating oxidative genes, Ppargc1a and Fgf21 in WAT (49). In BAT, cinnamaldehyde promoted the increase in the expression of thermogenesis markers, Ppara, Fgf21, and Ucp1. In rats, a long period of cinnamaldehyde treatment resulted in metabolic reprogramming, leading to a smaller WAT adipocyte size, accompanied by reduced expression of lipogenesis-related genes, Pparg and Dgat2; whereas in BAT, cinnamaldehyde led to reduced lipogenesis marker expression, Pparg and Lpl, associated with the reduced whitening, and an increase in Fgf21 expression (49).

Curcumin, or diferuloylmethane, is a flavonoid found in turmeric (*Curcuma longa* Linn.), a condiment used in Asian and Latin American cuisine. Early studies demonstrated the anti-diabetic and cholesterol-reducing properties of curcumin (50, 51). In addition, it was demonstrated that mice treated with curcumin presented body mass reduction, mostly due to increased energy expenditure (52).

**Figure 4:**
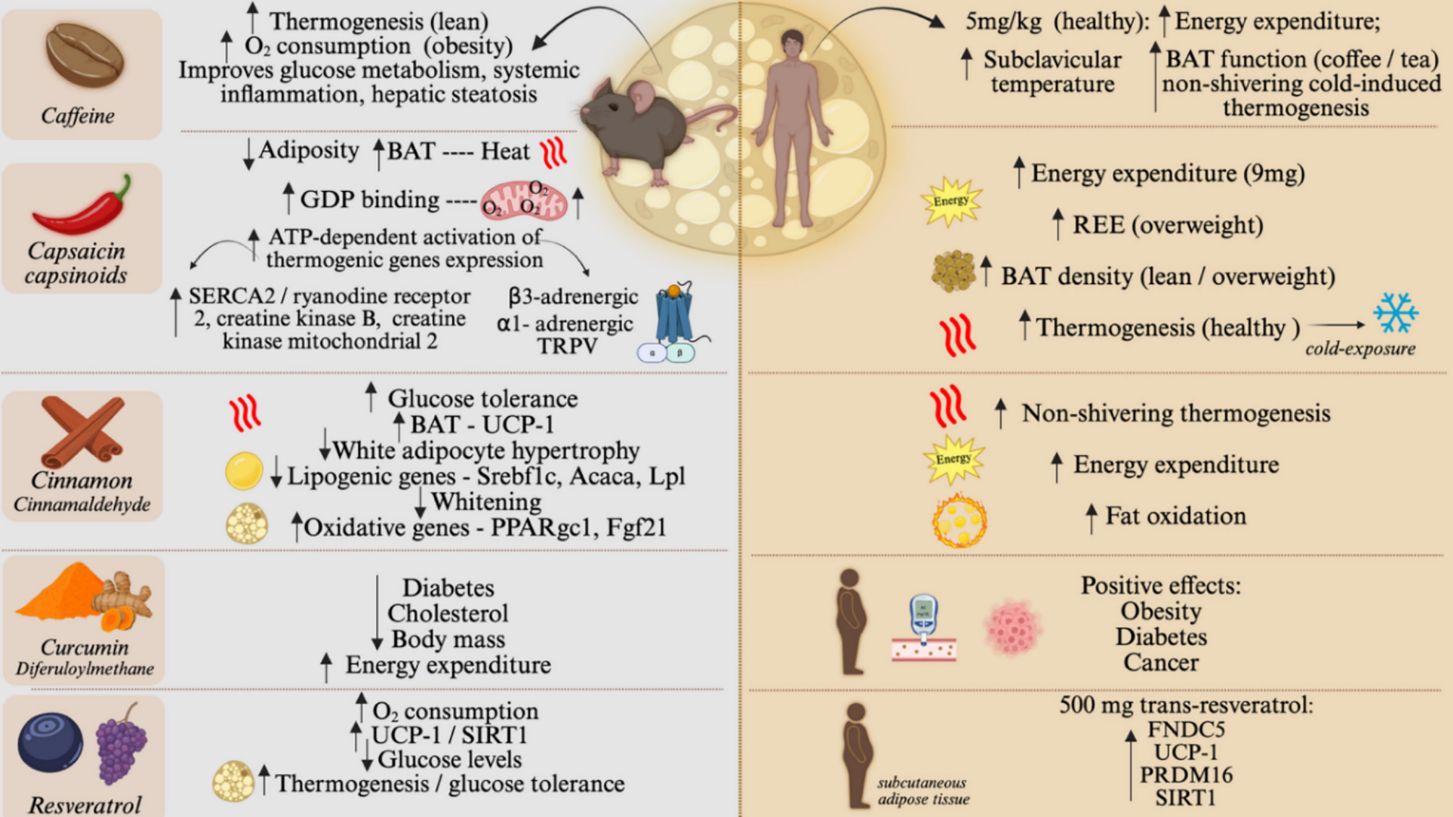
Food components effect on brown and beige adipose tissue, on animal models and humans. Effect of caffeine, capsaicin and capsinoids, cinnamon, curcumin, and resveratrol on BAT activity and browning of WAT. The left side of the figure shows the main results on rodents. The right side of the figure shows the main results of human studies. O2, Oxygen; SERCA2, sarcoplasmic/endoplasmic reticulum Ca2 + -ATPase 2; TRPV, Transient receptor potential vanilloid; SREBF1C, Sterol regulatory element-binding transcription factor 1; ACACA, Acetyl-CoA carboxylase alpha; LPL, Lipoprotein lipase; SIRT1, Sirtuin; REE, Resting energy expenditure; FNDC5, transmembrane protein fibronectin type III domain-containing protein 5; PRDM16, PR/SET domain 16; GDP, guanosine diphosphate; TRPV, Transient Receptor Potential cation channels; UCP1, Uncoupler Protein 1; PPARgc1, Peroxisome Proliferator-Activated Receptor Gamma, Coactivator 1; FGF21, Fibroblast Growth Factor 21. Created in BioRender. Velloso (2025) https://BioRender.com/jifpgbg.

Resveratrol, a natural polyphenol present in grapes and berries, has been studied for its role in activating brown/beige adipose tissue. In neonatal mice, the use of resveratrol reprogrammed the BAT toward a more thermogenic phenotype, and this was associated with changes in DNA methylation (53). In adult mice, resveratrol increased oxygen consumption, increased BAT expression of UCP1 and SIRT1, and reduced blood glucose levels (54). Even in monogenic extreme obesity due to the mutation of the leptin receptor (*db* mice), the treatment with resveratrol increased thermogenesis, activated the BAT, and attenuated glucose intolerance (55, 56). Figure 4 depicts the differential expression and role of each nutrient in mice and humans.

### The evidence for the thermogenic actions of food components in humans

Several clinical studies (Table 1) have shown the effects of dietary components on the activation of brown/beige adipose tissue. Capsaicin and capsinoids have become major players in this research field, as studies have shown a positive correlation of these compounds with metabolic health. In 2012, Yoneshiro and coworkers showed that middle-aged healthy men, who received an acute dose of 9 mg of oral capsinoids, had an increase in energy expenditure compared to the placebo group (57). The chronic use of capsinoid led to an increase in BAT density in normal and overweight men, as well as an increase in resting energy expenditure in overweight men (58, 59). It also increased cold-induced thermogenesis, measured by fluorodeoxyglucose-positron emission tomography and computed tomography, in healthy human subjects (60).

**Table 1:** Studies that tested the effects of nutrients or food components in human brown adipose tissue.

Nutrient-FC/DOI/Year	Title	Summary
Caffeine 10.1016/j.jtherbio.2021.103000 2021	Physically active men with high brown adipose tissue activity showed increased energy expenditure after caffeine supplementation	Human. IR camera. Capsule caffeine (375 mg vs. placebo) 30 min before data collection. BAT activation (temperature ROI) increased
Caffeine 10.1038/s41598-019-45540-1 2019	Caffeine exposure induces browning features in adipose tissue *in vitro* and *in vivo*	*In vitro* and *in vivo*. Adipocytes (1 mM caffeine) increased UCP1 and enhanced O2 consumption and proton leak + browning-like structural changes in mitochondrial and lipid content + increased thermogenic genes expression. Human: drinking coffee (vs water) stimulated temperature at supraclavicular region
Capsaicin 10.3390/nu12092676 2020	Effects of capsinoid intake on brown adipose tissue vascular density and resting energy expenditure in healthy, middle-aged adults: a randomized, double-blind, placebo-controlled study	Human. Capsinoid (9 mg/d vs. placebo) for 6w. Increased REE and BAT density
Cinnamon 10.1016/j.jep.2020.113413 2021	*Cinnamomum cassia* extract promotes thermogenesis during exposure to cold via activation of brown adipose tissue	Human and *in vitro*. IR camera. With cinnamon extract, increase in EE and body temperature, decreased lipid droplets and increased number of mitochondria, increased expression of thermogenic proteins
Ginger 10.1017/S0007114512005715 2013	Grains of paradise (*Aframomum melegueta*) extract activates brown adipose tissue and increases whole-body energy expenditure in men	Human. PET-CT FDG18 after 2 h cold (19°C) before ginger supplementation to determine BAT. After 4w crossover design, at RT, EE of BAT+ group increased
MUFA 10.1038/s41598-022-08125-z 2022	PET/MRI-evaluated brown adipose tissue activity may be related to dietary MUFA and omega-6 fatty acids intake	Human. Presence of BAT in subjects with lower consumption of omega-3 and MUFA
MUFA 10.1210/clinem/dgaa824 2021	Short dietary intervention with olive oil increases brown adipose tissue activity in lean but not overweight subjects	Human. 4w olive oil supplementation. Increased BAT activity in lean but not OW/obese, increased secretin, FGF21 and 12,13-diHOME
PUFA 10.1002/mnfr.201500892 2016	Eicosapentaenoic acid and arachidonic acid differentially regulate adipogenesis, acquisition of a brite phenotype and mitochondrial function in primary human adipocytes	Human preadipocytes were treated with w-3 (EPA) or w-6 (ARA). Day 12 of differentiation. EPA promotes browning and improves mitochondrial function and UCP1. ARA reduced mitochondrial respiratory function, increased lipid droplet size and white-specific marker
Resveratrol 10.1016/j.molmet.2018.04.004 2018	Resveratrol improves *ex vivo* mitochondrial function but does not affect insulin sensitivity or brown adipose tissue in first degree relatives of patients with type 2 diabetes	Men received resveratrol (150 mg/d) for 30d, cross over. Did not improve insulin sensitivity, BAT activity *FDG 18 + cold. *In vitro* resveratrol in human BAT adipocytes no effect
Tea Catechins 10.3945/ajcn.116.144972 2017	Tea catechin and caffeine activate brown adipose tissue and increase cold-induced thermogenic capacity in humans	Human. Acute effect (615 mg catechin or 77 mg caffeine beverage): increased EE with catechin. Chronic effect: catechin beverage 2x/d for 5w. 2 h cold (19C): increased EE

Caffeine has also been evaluated for its thermogenic actions in humans. Clinical studies using caffeine supplementation demonstrated positive effects on thermogenesis. A single supplement of caffeine capsules (5 mg/kg) resulted in an increase in energy expenditure and supraclavicular temperature, measured by infrared thermography, in healthy young men. The effect was seen 30 min after ingestion and lasted for 60 min (61). The consumption of coffee and tea, and beverages that contain caffeine, promoted BAT function in thermoneutrality, increasing energy expenditure, and non-shivering cold-induced thermogenesis (62, 63). Morbidly obese patients who received treatment with caffeine three times a day had more detectable β3-adrenoceptor in their adipocytes, revealing a potential mechanism of the action of caffeine on metabolic health (64).

Other nutritional compounds have been studied for their effects on non-shivering thermogenesis and metabolism. However, there is limited data on human volunteers. Resveratrol has also been studied for its potential thermogenic actions in humans, yet, the results are not as consistent as in experimental models. The chronic use of resveratrol (500 mg trans-resveratrol) increased thermogenesis markers, such as FNDC5, UCP1, and PRDM16, in obese human subcutaneous adipose tissue, as well as SIRT1 (Sirtuin 1). The activation of SIRT1 by resveratrol is hypothesized to be responsible for the positive effect of resveratrol on metabolism (65). Cinnamon has also been shown to influence non-shivering thermogenesis. In healthy subjects, the ingestion of the cinnamaldehyde resulted in an increased energy expenditure, fat oxidation, and nose temperature, after 90 min of ingestion. The positive effect of cinnamon is associated with the activation of the TRP channel, similar to capsaicin (66). Curcumin has also been evaluated for its thermogenic actions in humans. Studies have demonstrated that the consumption of curcumin can have a positive effect on human chronic diseases, such as obesity, type 2 diabetes, and cancer (67, 68). However, despite some promising results from animal models regarding curcumin’s role in non-shivering thermogenesis, there is a lack of human studies. This can be attributed to the poor bioavailability of curcumin formulations (69). Interestingly, other compounds from the ginger family, such as grains of paradise, have been studied in human subjects. Thus, it was observed an increase in energy expenditure, through the activation of BAT, 2 h after the oral administration of the compound to middle-aged men (70). In addition, it has been shown that the chronic use of grains of paradise by non-obese, young, female volunteers led to reduced visceral fat and elevated energy expenditure (71).

### The evidence for the thermogenic actions of nutrients in experimental models

As fatty acids are the main substrates for uncoupled mitochondrial respiration and thermogenesis both in brown and beige adipocytes, it was expected that increased dietary consumption of such nutrients could stimulate adaptive thermogenesis. There is fine experimental evidence that mono-, and polyunsaturated fatty acids (MUFAs and PUFAs, respectively) can act through distinct mechanisms to activate BAT and beige adipose tissue. Eicosapentaenoic acid (EPA) can act directly in isolated brown adipocytes inducing the expression of thermogenic genes through the activation of the fatty acid receptor GPR120 and intracellular signaling through cAMP (72). In mice, both EPA and docosahexaenoic acid (DHA) given as dietary supplementations increase thermogenesis in both BAT and beige adipose tissue by promoting the direct activation of GPR120 and TRVP1 (73, 74). Moreover, both EPA and DHA can also act in the central nervous system, stimulating thermogenesis through the activation of the sympathetic nervous system (75). The actions of EPA and DHA in the brain to control thermogenesis occur, at least in part, through the activation of hypothalamic GPR40 and GPR120 (76). GPR40 is expressed in pro-thermogenic proopiomelanocortin (POMC) neurons in the hypothalamus, and the treatment with either DHA or a synthetic agonist results in an increase in whole-body energy expenditure and activation of BAT (77, 78).

The MUFA, oleic acid, has also been shown to stimulate thermogenesis. A study showed increased oxygen consumption and thermogenesis in rats fed on a diet rich in oleic acid as compared to those fed predominantly on saturated fats (79). Another study using mice compared a control diet containing 5.6% kcal fat from lard and 4.4% kcal fat from soybean oil with high-fat diets containing 25% kcal from lard and 20% kcal fat from either shea butter (stearic acid-rich fat; SHB), olive oil (oleic acid-rich oil), safflower oil (linoleic acid-rich oil), or soybean oil (mixed oleic, linoleic, and α-linolenic acids). The results showed that the diet containing oleic acid was the most efficient in mitigating body mass gain, which was accompanied by increased thermogenesis, increased BAT expression of UCP1, and improved glucose tolerance (80). Oleic acid supplementation in rats was also shown to be as effective as exercise to induce thermogenesis, at least in part by stimulating the trans-differentiation of white to beige adipocytes (81). A recent advance in the field was obtained by the identification of GPR3 as the receptor for oleic acid. In GPR3 knockout mice, oleic acid fails to increase BAT thermogenesis, whereas in intact mice, it triggers an intracellular signaling cascade activating Gs/cAMP and PKA (82).

### The evidence for the thermogenic actions of nutrients in humans

Different from experimental data, the actual impact and direct actions of dietary fats and other nutrients on the activity of human BAT and beige adipose tissue are still controversial (83, 84). For example, the level of evidence is much smaller than the data showing that MUFAs and PUFAs have anti-inflammatory and cardiovascular protective actions (85, 86). Nevertheless, there are some interesting data (Table 1) that reinforce the need for further studies that could provide consistent advances in the field.

In a clinical study, EPA was shown to promote mitochondrial biogenesis in subcutaneous adipocytes of overweight individuals through up-regulation of mitochondrial transcription factors NRF-1 (nuclear respiratory factor-1) and TFAM (mitochondrial transcription factor A), increased activity of SIRT1 (Sirtuin1), deacetylation of PGC-1α (Peroxisome Proliferator-Activated Receptor γ Coactivator 1α) and increased phosphorylation of AMPK (AMP-activated protein kinase), an energy sensor responsible for stimulating fatty acid oxidation and activating PGC-1α. The authors suggested the role of EPA in remodeling adipocyte metabolism, inducing greater efficiency in the use of fatty acids as energy fuel, in addition to the positive regulation of beige adipocyte markers by increasing the expression of PRDM16 (PR domain containing 16), CIDEA (cell death-inducing DFFA-like effector a) and UCP1 (uncoupling protein 1), genes involved in WAT beiging (87).

Another clinical study has revealed that diet-induced thermogenesis was more pronounced after consuming PUFA-enriched sources as compared to saturated fatty acids (88). Furthermore, it has been shown that PUFAs can mitigate obesity-associated BAT mitochondrial dysfunction by regulating thermogenic gene expression (89). In a 4-week MUFA intervention, the BAT activity was increased in lean but not in obese volunteers, suggesting that optimal thermogenic action promoted by dietary unsaturated fats depends on preserved insulin sensitivity in the BAT (90).

Another interesting trial with healthy young men showed that 12-week daily supplementation with 2 g EPA and 1 g DHA increased the oxidation of fatty acids and carbohydrates only under cold environmental conditions, regardless of basal metabolic rate. This suggests that PUFA-mediated thermogenic activation may be dependent on cold environmental stimuli (91).

An important advance in the field was provided by a study that evaluated the lipidomic profile of humans and mice and observed that β3-adrenergic stimulation through cold exposure promoted the activation of the enzyme 12-lipoxygenase (12-LOX), which has as a by-product, the lipid derivative 12-hydroxy eicosapentaenoic acid (12-HEPE), an important paracrine and/or endocrine factor that captures glucose in BAT and muscle, improving the efficiency of this signaling cascade in glucose metabolism (80). This is important because it shows that a substance derived from nutrients and produced in the BAT can play a role in the regulation of thermogenesis. This will be explored in the next section.

## Nutrient-derived substances that regulate thermogenesis

12,13-diHOME (12,13-dihydroxy-9Z-octadecenoic acid) is an oxidized linoleic acid metabolite that is produced by the BAT. In 2017, Lynes and colleagues explored the correlation between 12,13-diHOME and cold in both humans and mice. They showed that, in humans, blood levels increased after cold exposure and it was negatively correlated with BMI, insulin sensitivity, and triglycerides. In mice, a pre-treatment with 12,13-diHOME increased cold tolerance and increased fatty acid uptake in brown adipocytes by stimulating fatty acid transporters, CD36 and FATP1 (92). Moreover, another study showed that 12,13-diHOME was inducible by exercise both in humans (female, male, young and old) and in mice, and increased the fatty acid uptake in skeletal muscle and mitochondrial respiration showing the role of exercise, in addition to cold, to increase 12,13-diHOME levels (93). In mouse models, this adipokine is also related to a reduced inflammatory scenario and reduced atherosclerosis by acting on endothelial cells and enhancing eNOS (94).

**Figure 5:**
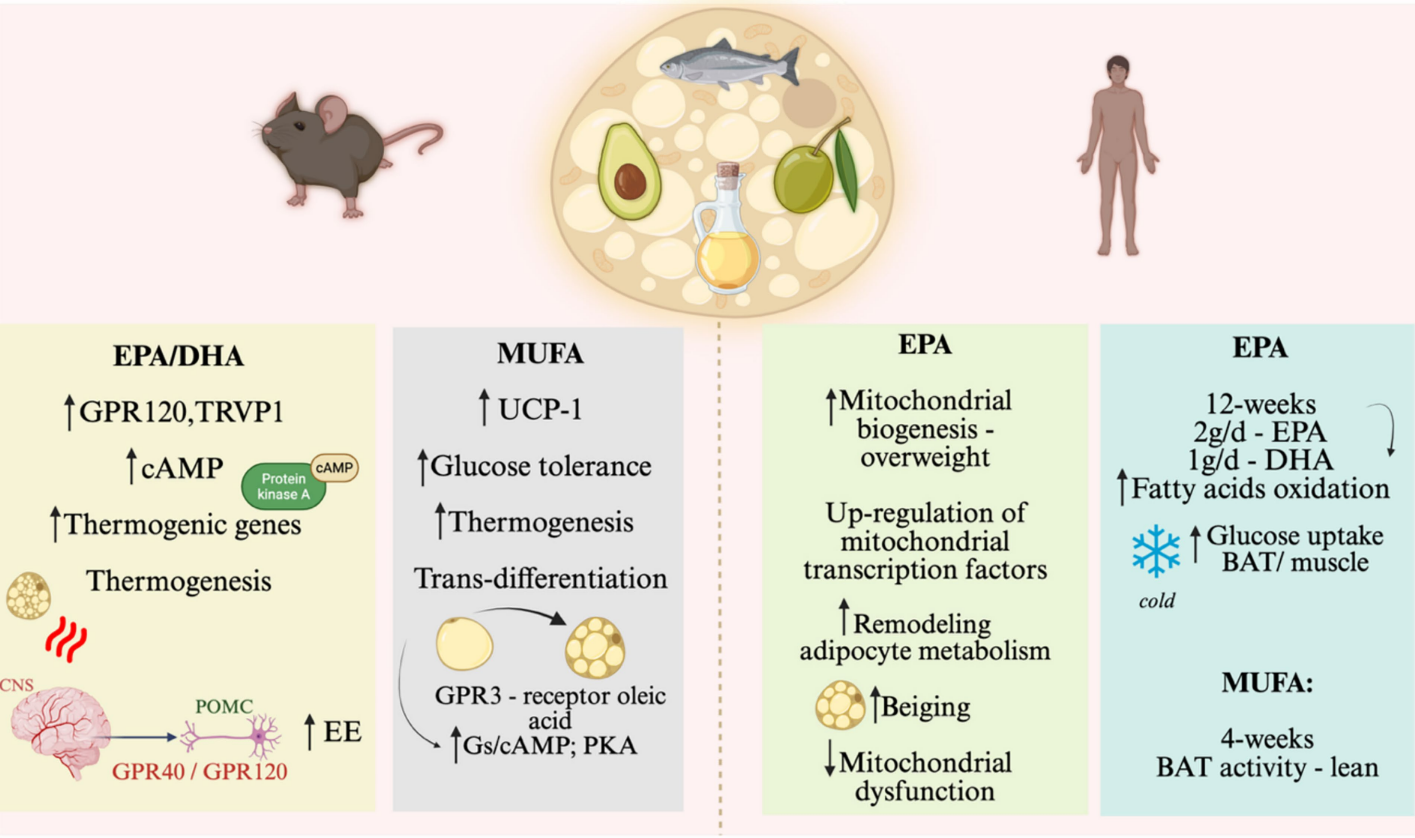
Nutrient effects on brown and beige adipose tissue, on animal models and humans. Effect of EPA/DHA and MUFA on BAT activity and browning of WAT. The left side of the figure shows the main results on rodents. The right side of the figure shows the main results of human studies. EPA, eicosapentaenoic acid; DHA, Docosahexaenoic acid; GPR, G-protein coupled receptor; EE, Energy expenditure; MUFA, Monounsaturated fatty acids; Gs/cAMP, stimulatory G protein that produces cyclic AMP (Adenosine monophosphate); POMC, proopiomelanocortin; GPR40, G protein receptor, also known as FFAR1 (Free Fatty Acid 1); GPR120, G protein receptor, also known as FFAR4 (Free Fatty Acid 4). Created in BioRender. Velloso (2025) https://BioRender.com/bxkid6u.

Another class of compounds related to metabolism is oxylipins, produced by oxygenases that oxidize PUFAs. In the BAT, one of these enzymes, the 12-LOX, is responsible for the release of 12-HEPE, an oxylipin, in response to cold. Acting in brown adipocytes, 12-HEPE increases the expression of *de novo* lipogenesis genes and Glut1 and promotes the translocation of GLUT4 to the cell membrane, increasing glucose uptake and stimulating thermogenesis. This mechanism was shown to occur through the activation of the PI3K-mTOR–Akt-Glut pathway (95). Lipoxygenases like 12-LOX are also involved in response to inflammation and are widely expressed in many tissues, so the BAT-specific roles of its products are still under investigation. 12-HEPE circulating levels were increased in patients with severe obesity compared to healthy volunteers; however, the blood levels of 12-HEPE 1 year after sleeve gastrectomy were reduced, perhaps as a consequence of a reduction in the body amounts of PUFAs (96). In a human randomized, double-blind controlled, crossover study with volunteers with obesity, 12-HEPE was inversely correlated with serum IL-10, an anti-inflammatory cytokine but it was also inversely correlated with IL-6 and MCP1 (97). Further studies are needed to provide advances in the understanding of the roles of 12-HEPE in immune response. Figure 5 depicts the effects of fatty acids on mice and human brown adipose tissue as presented in this subsection.

## Micronutrients and brown adipose tissue activity

Vitamins and amino acids have also been recently studied to address their potential role in the regulation of thermogenesis and metabolism. In a recent review, vitamin A was shown to modulate the activity of the brown adipose tissue. Retinol is a potent transcriptional factor that regulates pathways important in adipose tissue thermogenesis. In adipocytes, retinol is converted to retinaldehyde and retinoic acid, which promote the expression of PGC1alpha and UCP1 (98). In mice, the treatment with retinoic acid enhanced UCP1 expression in BAT depots and prevented the whitening of the tissue (99). In another study, mice treated subcutaneously with retinoic acid exhibited increased expression of thermogenic markers in white adipose tissue, even at thermoneutrality (100). Moreover, retinaldehyde, a retinol precursor, was capable also of inducing the expression of thermogenic genes in human white adipocytes (101).

The impact of nutrients in BAT was also reviewed in a paper by Noriega and colleagues (102). They discuss the role of vitamin D in brown adipocytes and thermogenesis. In human neonates, vitamin D can increase mitochondrial biogenesis in beige adipocytes (103). In mouse models, vitamin D seems to exert its potential as a thermogenic activator in dose-dependent manner. Its deficiency is associated with reduced expression of UCP1 and an obese phenotype in rats (104). In mice knockout for the vitamin D nuclear receptor (VDR) or the enzyme responsible for converting vitamin D to its active form, there was an increase in body weight gain (105).

In another recent and excellent work (106) about browning in human fibroblast-derived adipocytes, the authors highlight the importance of the amino acid choline, and vitamin B pantothenic acid, on the expression of UCP1 and mitochondrial respiration in human brown adipocytes. They demonstrate that small amounts of pantothenic acid induce UCP1 expression, increase lipolysis and oxygen consumption by the cells. Choline and pantothenic acid are crucial and necessary for the development of a browning phenotype during human fibroblast differentiation into brown adipocytes. However, high doses of pantothenic acid have the opposite effect and reduce glycolysis. Riboflavin (B2), another vitamin B, increases, in a dose-dependent manner, lipolysis but reduces UCP1 expression and mitochondrial membrane potential. Thiamine (B1), yet another member of the vitamin B family, increases UCP1 but with no effect on membrane potential and reduces lipolysis.

Iron is another micronutrient that participates in the correct functioning of mitochondria and consequently on thermogenesis. Brown adipocytes require more iron than white adipocytes due to mitochondrial biogenesis and reduced iron availability suppress brown and beige adipocytes differentiation (107). In mice, iron deficiency in association with the consumption of HFD, impaired insulin metabolism, increased weight gain and reduced body temperature control under cold exposure compared to adequate iron amount group of mice (108). Similar to the dose-dependent action of vitamin B, high-iron diet exacerbates the effects of HFD in mice, exhibiting impaired insulin signaling, glucose metabolism, reduced brown adipose tissue activity and higher mitochondrial oxidative stress (109). Thermogenesis requires appropriate sympathetic innervation to work properly, and zinc participates in this scenario regulating this adrenergic input. In obesity, reduced zinc impairs this innervation while its supplementation ameliorates thermogenesis (110). An important point to highlight is that scientific evidence of the role for these micronutrients on thermogenesis is usually designed exploring deficiency or supplementation and rarely with standard meal amounts or food as main source pointing the relevance of continuous studies in this field aiming human improvement on metabolism with browning and thermogenesis approaches.

## The impact of changes in the activities of brown and beige adipose tissues on health and disease

Changes in the activity of brown and beige adipose tissues have a considerable impact on health and disease, particularly regarding metabolism and energy balance. Increased activity of these tissues can lead to increased energy expenditure, improved glucose and lipid metabolism, and better control of body weight and metabolic parameters. Conversely, reduced activity of BAT and beige, or the predominant presence of WAT, is associated with obesity, insulin resistance, and other metabolic abnormalities (111).

One of the greatest advances in the field was achieved by the evaluation of the presence of active BAT in more than 134 thousand ^18^F-FDG positron emission tomography-computed tomography (PET/CT) reports from more than 50 thousand patients (2). The study confirmed two important features of the BAT showing that its activation is inversely proportional to environmental temperature and directly proportional to body mass index. In addition, it was revealed for the first time that the presence of active BAT protects against type 2 diabetes, dyslipidemia, coronary artery disease, cerebrovascular disease, congestive heart failure, and hypertension (2). The subjects evaluated in the study were not under specific interventions aimed at modulating the activity of BAT; thus, they can be regarded as people who presented active BAT under regular conditions of life. In the future, it will be important to determine if people who present increased BAT activity as a consequence of a specific intervention, including food interventions, also benefit from the protection against such diseases.

Sex, age, and ethnic differences are important and yet underexplored issues in BAT and beige adipose tissue biology. In a retrospective study evaluating PET-CT scans of over 5,000 subjects, females presented greater positivity than males, and, in general, BAT positivity was reduced with aging (2). Regarding ethnicity, it has been shown that South Asians tend to have smaller BAT volumes compared to white North Americans and Europeans, even when controlling for factors like BMI and body fat percentage (112). Moreover, African ancestry seems to be associated with reduced BAT activation in response to cold exposure (113). It is yet unknown how these distinct groups respond to nutrients and food components as interventions aimed at modulating BAT and beige adipose tissue activity.

## Conclusion

**Figure 6:**
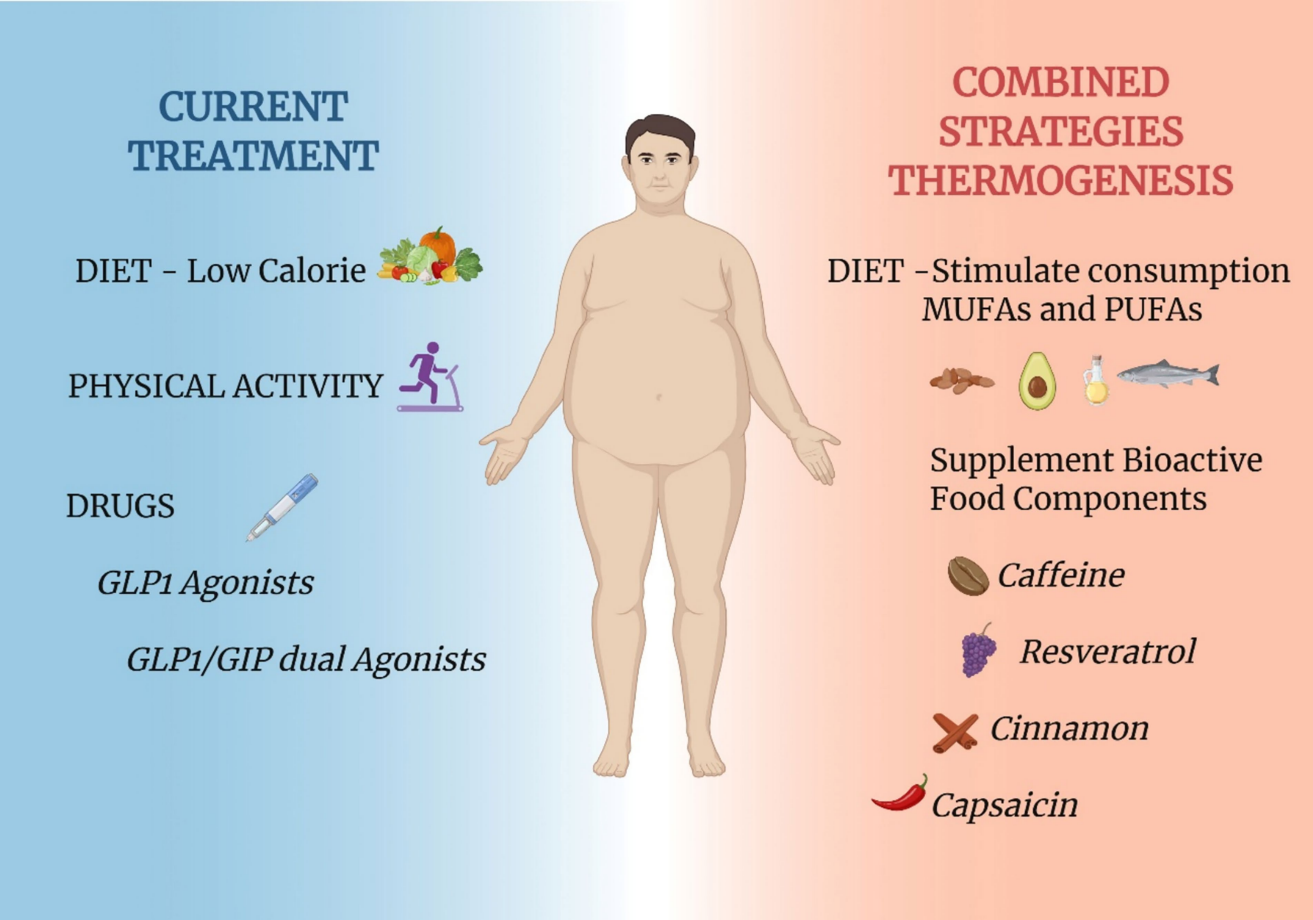
Proposed combinatorial strategies to improve thermogenesis. Currently, obesity treatment guidelines recommend the association of dietary intervention, increased physical activity and use of pharmacological agents that reduced caloric intake (left-hand side of the panel). Considering the current and future understanding of the impact of certain nutrients and food components as thermogenic agents, a putative combinatorial strategy could result in increased body mass reduction. The combinatorial strategy could rely on the recommendation for increased consumption of MUFAs and the supplementation with thermogenic bioactive food components (right-hand side of the panel). This proposal could be considered in future clinical trials aimed at advancing the management of obesity. Created in BioRender. Velloso (2025) https://BioRender.com/6qh7u9t.

The development of agonists of the GLP-1 receptor has brought remarkable advances in the treatment of obesity and diabetes (114). In clinical trials, body mass reductions approached the magnitude previously achieved by bariatric surgery, only (115). Early experimental data suggested that the results on body mass reductions were due to a combination of reduced caloric intake and increased energy expenditure (116). However, a detailed mechanistic evaluation of the actions of semaglutide, refuted the thermogenic effects, suggesting that at most, it could mitigate the drop in energy expenditure that accompanies body mass reduction (117). At first, these findings were interpreted as a therapeutic weakness of the GLP-1 receptor agonists. However, it can also be seen as a new window of opportunity. Considering that weight reductions of 20–25% can be achieved by a class of drugs that act on caloric intake, only, what would be the outcomes of a combined use of a GLP-1 receptor agonist plus a drug that increases thermogenesis? In experimental studies, this has been tested, and the results are promising. The combined use of liraglutide and succinate, an intermediate of the Krebs cycle that acts directly in BAT mitochondria to stimulate thermogenesis, resulted in increased body mass reduction (118). The combined use of liraglutide plus an agonist of the fatty acid receptor, GPR40, also resulted in increased body mass reduction (77). Thus, there is considerable experimental evidence suggesting that body mass reductions greater than 20–25% are feasible. Some of the potential targets for the development of thermogenic drugs may come from studies that explored the thermogenic actions of nutrients and food components. Moreover, there is also much to be done by exploring the combination of drugs that reduce caloric intake and dietary patterns that increase thermogenesis. In this review article, we have put together experimental and clinical data that provide evidence for the thermogenic actions of several nutrients and food components. Further clinical studies should analyze the impact of combining state-of-the-art pharmacological interventions and nutrients/food components as strategies to increase body mass reduction and metabolic control (Figure 6).

In conclusion, there is growing evidence that some nutrients and food components possess thermogenic capacity. Exploring these properties has led to the identification of potential molecular targets for drug development (119). Moreover, the refined exploration of the thermogenic actions of certain foods could lead to changes in the dietary recommendations for patients with obesity, diabetes, and other metabolic and cardiovascular conditions.
